# Development of effective sequence multi-barrier reactive media for nitrate remediation in groundwater systems

**DOI:** 10.1039/c8ra10669j

**Published:** 2019-05-17

**Authors:** Muntaka Dahiru, Abu Bakar Nor Kartini, Ismail Yusoff

**Affiliations:** Department of Chemistry, University of Malaya 50603 Kuala Lumpur Malaysia kartini@um.edu.my gwanitahir@gmail.com; Department of Geology, University of Malaya 50603 Kuala Lumpur Malaysia ismaily70@um.edu.my; Department of Science Lab. Tech., Kano State Polytechnic Nigeria

## Abstract

Early depletion of carbon-based electron donors and pollution swapping are among the recalcitrant challenges for *in situ* remediation technology. There is a pressing need to provide a slow but steady dose of materials for a prolonged period in order to sustain *in situ* treatments. In an effort to develop effective sequence multi-barrier reactive media that can address these challenges, the present study reports laboratory column experiments using organic (date seed, *Moringa oleifera* and wood chips) and inorganic (limestone; CaCO_3_) carbonaceous materials in varying proportions. The substances are supported by paleo sandy beach soil and are sequentially packed in line with the geometric pattern of a hypothetical funnel and gate for nitrate (NO_3_^−^) remediation. The optimized reactive media show remarkable efficiency, remediating 94.4% of NO_3_^−^ as a target pollutant within the system. They also remediate 99.20% of the NO_3_^−^ down the gradient and 96.4% of its lethal by-product – NO_2_^−^. However, with the generation of an 11-fold biomass build-up, the system is only able to eliminate 53% of the total organic carbon generated. Hence, the advective dispersive features of aquifers are required for the complete remediation of the generated secondary biomass. The results also reveal that the efficiency of the sequential media depends on the composition/ratio of the constituents, flow rate and the initial concentration of NO_3_^−^ in the influent. Surface characterization studies (FESEM-EDX and FTIR) show that compared to wood chips, date seed is less prone to degradation, suggesting a longer life span. A degradation study affirms the findings of the surface studies as it reveals that the half-life of date seed is three times longer than that of wood chips. In addition, the study reveals that the double exponential model is more suitable than the single exponential model in determining the rate of decay of both wood chips and date seed.

## Abstract 2

Using surface characterization and a decomposition study, carbonaceous materials are found to have longevity potential in novel reactive media for *in situ* denitrification.

## Graphical abstract

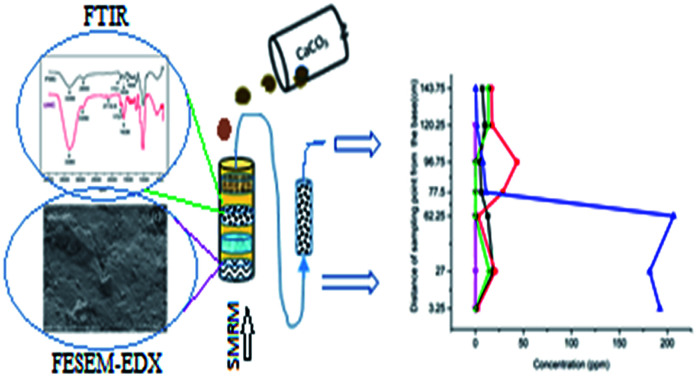

## 1 Introduction

The threat of groundwater nitrate contamination has been raising global concerns as human activities that emit both point and non-point sources of pollution are increasing at an alarming rate. The European Union water framework once directed that, for safety purposes, the increasing anthropogenic groundwater contamination must be reversed by 2015. Anthropogenic inputs to nitrate accumulation in groundwater have been rated as high, with agricultural activities accounting for 45% of nitrate accumulation.^1^ Considering the effect of nitrates on human health and the reliance of a large number of people on groundwater exploitation, nitrate-polluted water remediation is attracting growing attention. Currently, a biological remediation technique using a permeable reactive barrier (PRB) has been identified as the most cost effective and safe technique.^2^ However, the technology is facing several challenges, which limit its application for site-specific reasons. Site-specific parameters such as groundwater flow rate, mineral content, uniformity of packing structure (heterogeneity or homogeneity), pH variation pattern,^3^ dissolved oxygen content and availability of electron donors (such as organic carbon, Fe and Mn) are reported to significantly influence the performance of the reactive barrier.^4^

A PRB is a subsurface structure that can either be in the form of continuous wall or a funnel and gate. The gate is the permeable site, packed with reactive media for remediation. Factors such as the influence of the packing structure,^5^ composition of the reactive media,^3^ positioning of the gate in relation to the impermeable wall,^6^ mechanism of remediation, and nature and state of the reactive media have great influence over the efficiency of remediation at the gate.^7^

In the biological remediation technique, the use of natural substances (such as carbon sources) in the PRB gate for denitrification is very useful as bacteria in subsurface-saturated zones can obtain energy from both organic and inorganic sources. Examples of carbon sources used in field and laboratory scale remediation include methanol, ethanol, sucrose, glucose,^8,9^ soft wood, pine bark, sawdust and plant compost.^5,10,11^

In a natural setting, both heterotrophic and autotrophic denitrifications occur simultaneously.^12^ Heterotrophic denitrification is observed to be the dominant process. It also facilitates other forms of denitrification by depleting oxygen. However, heterotrophic denitrification is limited by the rapid decline of the carbon source at the gate.^13^ Therefore, there is a need to develop techniques that will ensure the consistent, slow and stable release of carbon from cellulose-based solid substances.^5^ A similar paradigm shift of strategy is taking place in remediating chlorocarbon compounds where control release materials are being studied.^14^

Safety and long-term performance are also major concerns for PRBs. In addition, pollution swapping often occurs in which the by-products from the decomposed reactive media generate other pollutants of concern. These problems have been preventing the smooth application of the technology. While striving to provide solutions, field and laboratory studies (column studies) have been conducted with a number of proposed reactive materials in simulated aquifer conditions. Remediation assessments of petroleum products, nitrate, chlorinated hydrocarbons, metals in acid sulfate terrain and long-term performance have been conducted.^5,15^

In an effort to simulate the nitrate attenuation pattern, the present study reports a laboratory column experiment designed to fit into the gates of a novel twin-funnel and gate structure (unpublished work) in order to address the problem of early depletion of reactive media (longevity) and pollution swapping. This study, unlike many in the field, pays special attention to the type and nature of the soil, which was investigated to support heterotrophic and autotrophic denitrification. For the fast and steady supply of electron donors, some organic/inorganic substrates (date seed, *Moringa oleifera* seed and limestone) were utilized in different positions and in different proportions, representing multi-sequence reactive media.

The objective of this laboratory-scale column study was to evaluate the longevity performance of a combined biological and physical system of remediation. The study also tested a designed multi-sequence reactive barrier in line with a funnel structure, and the reliability of the permeable reactive interceptor (PRI) concept by evaluating the influences of the relative positions and proportions of the reactive media on the efficiency of remediation. This was achieved through studying the simultaneous mitigation of the main target pollutant (nitrate) and the released by-product of the organic sources (secondary biomass) within the media (in the first column) and down the gradient (in the second column). To the best of the authors' knowledge, this study is the first of its kind in terms of utilizing paleo-beach soil as the source of the inorganic electron donor (Fe) for autotrophic denitrification. It is also noteworthy that the arrangement of gate simulation in a column followed by downgradient aquifer simulation in a second column is essential for the progressive attainment of remediation goals.

## 2 Materials and methods

A combination of organic and inorganic electron sources was incorporated into the reactive media. These included date seed, sawdust, wood chips, *Moringa oleifera*, limestone and iron rich paleo-beach soil. The method of parallel-packing the structures in a series column arrangement was employed.

The reagents used included multi-element ion chromatography anion standard solution (certified reference material, Sigma-Aldrich) and, in line with the manufacturer's requirements, eluents consisting of 0.5 M NaCO_3_ and 0.5 M NaHCO_3_ as well as AnalaR grade KNO_3_ (Fisher Scientific). The instruments used included a Dionex 1100 IC, ultrasonic machine, digital multi-use lab shaker (Orbital Shaker), weighing balance, peristaltic pump (Masterflex L/S), desiccator, SSM-5000A Shimadzu total carbon (TOC) analyzer, Field Emission Scanning Microscopy-Energy Dispersive X-ray (FESEM-EDX) analyser (Hitachi, model: SU8220) and Fourier transform-infrared (FTIR) spectrophotometer (Spectrum 400).

### 2.1 Reactive media (materials) used and rationale

Based on the findings in a published work that led to this research, date seed was chosen because it had the lowest rate of water absorption capacity and least objectionable organic extracts of a range of seeds tested.^16^ In addition, the thick walled endosperm of the seed consists of over 90% mannan and a small amount of cellulose, serving as a store for carbohydrate.^17^ Moreover, date seed has been reported to resist decomposition for more than 2000 years.^18^ Other carbon sources selected for ease of decomposition and specific substrates of interest included humus (in the soil), *Moringa oleifera* (containing sulfate), sawdust, limestone and wood chips.

*Moringa oleifera* was used because the participation of sulfate reducing bacteria and the possibility of rapid reaction between undissociated protons (in H_2_S) and iron-derived electrons (for production of hydrogen) is well documented in the subsurface aqueous region. Therefore, the occurrence of hydrogenotrophic denitrification is highly likely.^19^ Moreover, the presence of metal sulfide in the form of iron sulfide in paleo-beach soil has been reported.^20,21^ It has also been reported that in coastal regions, H_2_S might be produced from respiratory reduction of sulfate from seawater (eqn (1)).^19^

1 Fe + H_2_S → FeS + H_2_

Lastly, a commercially available activated carbon was used for the adsorption of organic degradation by-products.

### 2.2 Preliminary treatment of the gate materials

In this work, the seeds of all the carbon sources were crushed to irregular shapes with sizes ranging from 3 mm to 5 mm. They were then washed with deionized water (to remove impurities) and screened for leachable nitrate and excessive organic acid using ion chromatography.^16^ The soil composite was applied to provide optimum permeability, Fe-based minerals and support. The percentage compositions of the materials were gravimetrically measured after considering the density of each material.

### 2.3 Experimental design and column set-up

The packing structure of the reactive media in the column was designed to simulate the cross-sectional area of a novel funnel and gate set-up as well as a pristine aquifer region down the gradient. Hence, two opaque PVC columns (first and second) with internal diameters of 4.9 cm were used. The heights of the first and second columns were 100 cm and 50 cm, respectively. The first column was set to simulate the reactive media while the second column simulated the attenuation pattern at the immediate aquifer region after the PRB. Water was pumped through both columns in an up-flow mode using a multiport peristaltic pump. In addition, a separate control column was filled with soil only.

**Fig. 1:**
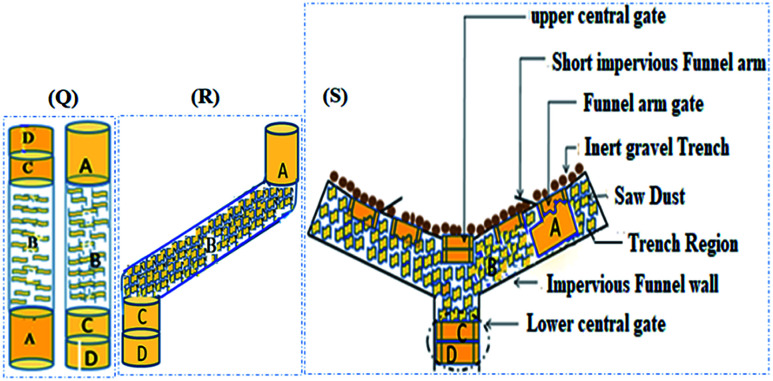
Order of the sequence multi-barrier reactive media in the funnel and gate. (Q) the column packing structure, (R) the alignment of the column packing structure with respect to the geometry of the funnel and gate, and (S) the funnel and gate. A, B, C and D are compartments as described.

As shown in Fig. 1, from bottom up, the first compartment (A) consists of an 18 cm long mixed layer packed with *Moringa oleifera*, soil, date seeds, and limestone in the ratio of 1.0 : 115.4 : 50.5 : 24.3. The second layer (B) is 30.5 cm long, and consists of a mixture of soil, wood chips, sawdust and limestone in the ratio of 32 :  3: 1 : 5.2. The third (C) and fourth (D) compartments constitute 35% of the total column height. The third layer (10 cm long) consists of a mixture of soil, date seed and limestone in the ratio 8.3 : 1.7 : 1, while the fourth (13.5 cm long) consists of activated carbon and soil in the ratio 1 : 1.6. The pore volume and porosity were measured to be 693 cm^−3^ and 43.6% respectively. From bottom up, five sampling points (P) were punctured along the column height at 3.25 cm (P_1_) (influent), 27 cm (1^st^ layer-P_2_), 62.5 cm (2^nd^ layer-P_3_), 77.5 cm (3^rd^ layer-P_4_) and 96.75 cm (4^th^ layer-P_5_). In both columns, water samples were abstracted from the influent port, three intermediate sampling ports (P_2_ to P_4_) and the effluent port (P_5_) for analysis.

To attain a stable flow and acclimatization, the column administered with distilled water was run for three weeks before nitrate-spiked groundwater was loaded.^22^ For the same reason, the nitrate-spiked groundwater in all three phases was administered for 2 days to allow for an adequate incubation period before sampling for analysis.^23^ In addition to the adjusted material composition, the operating flow rates for running the nitrite-spiked distilled water and well water columns were 3.15 mL min^−1^ for the first, second and third runs and an adjusted flow rate of 2.5 mL min^−1^ for all of the other runs. The experiments were run at ambient temperature and the average pH values in the final run were 7.1 and 7.6 for the inlet and outlet sampling locations, respectively.

#### 2.3.1 Alignment of the column packing structure with the PRN design

Unlike most previous studies, this study attempted to align the packing structure of the gate with the funnel configuration. The layers are represented with letters A to D as shown in Fig. 1. During the column analysis, the order is reversed since the influent was loaded from the bottom up (Fig. 1, Q).

### 2.4 Analytical methods and instruments

Dynamic parameters such as the concentrations of PO_4_^3−^, Cl^−^, SO_4_^2−^, NO_3_^−^ and NO_2_^2−^ were measured using ion chromatography while the temperature and pH were measured with a Mettler Toledo pH meter.

Instrument validation was done by running intra-day and inter-day analyses in triplicate; the percentage deviation was 2% (*i.e.* 98% measurements were within the range). Total organic carbon (TOC) was measured using the TOC analyzer. Volumetric and gravimetric methods were employed to measure the pore volume and porosity of the packed columns. Differences in the morphologies and functional group appearances between fresh and used hard carbon sources (date seed and wood chips) were explored for interpreting the FESEM-EDX and FTIR results in order to predict longevity potential and degradation patterns of the reactive media. The analytical weighing balance, ovum and desiccator were used for the gravimetric determination of mass loss using the litter bag technique.

#### 2.4.1 The rate of decomposition

The mesh bag technique^24^ was employed to determine rates of decomposition, with adjustments on tagging and distribution. Date seeds and wood chips were dried at 60 °C for 48 hours before usage. Efforts were made to cut the date seeds into two equal halves (by size and by weight). Combined date seeds with a total mass of 1 g were chosen for the degradation study while the endosperm was carefully scratched off the complementary halves to leave only the hard cover of the seeds, and the weight of each portion was recorded to assess the endosperm content. The mass of wood chips used for this work was approximately 1 g. To assess the influence of the nutrients on the degradation pattern of the carbonaceous solids, the same treatment was applied to controlled samples, which were introduced into a column packed only with soil, and fed with un-spiked groundwater. The procedure of Wider and Lang^24^ was followed for the litter analysis, and the technique of Fisher^25^ was used for determining mass loss. The rate of decomposition was determined using a single exponential model and a double exponential model.^26,27^

## 3 Results and discussion

The results of the column analysis, surface characterization and decomposition of the materials proved the efficacy of the gate material and provided an insight into the decomposition pattern of the carbonaceous material used in the experiment.

### 3.1 The column experiment

Considering the observed denitrification potential of the soil^28,29^ used in this experiment, the soil was envisaged to provide the denitrifying species in the system. The column experiment was divided into three phases.

#### 3.1.1 Phase I – distilled water

**Fig. 2:**
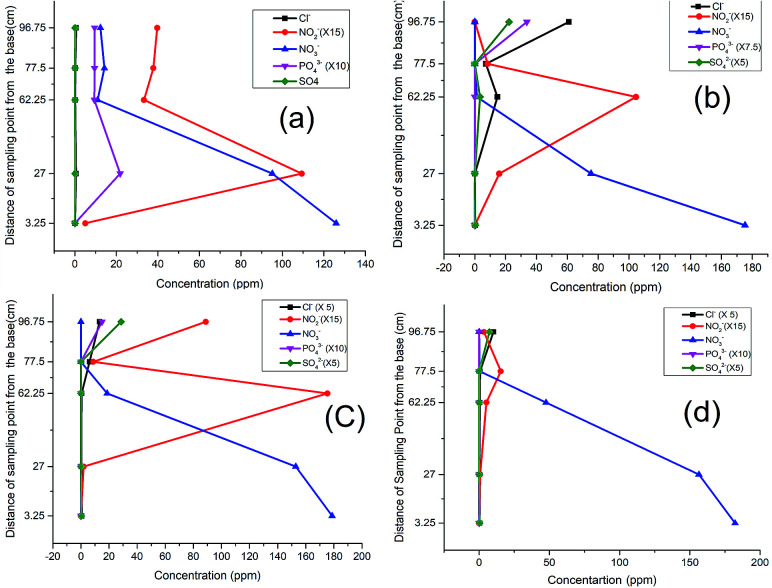
Response of NO_3_^−^ spiked distilled water: (a) 1^st^ run, (b) 2^nd^ run, (c) 3^rd^ run, (d) 4^th^ run.

When the nitrate-spiked distilled water was run through the column, the pattern of nitrate and nitrite attenuation in the column indicated a rapid nitrite buildup, attaining its peak at P_2_, after which the nitrate concentration dropped sharply reaching its lowest value at P_3_ (Fig. 2a). This sudden response may be explained by the revitalizing presence of the nutrient (NO_3_^−^) in the degradation of the carbon material by the microbes, leading to an incomplete denitrification process, which tended to attain some level of relative stability after the second layer at P_3_.

As the test continued in the second run (Fig. 2b), a clear shift in the nitrite peak was observed at P_3_ suggesting the acclimatization of the microbial community and active engagement of the sawdust chamber. A noticeable rise of peak for chloride, sulphate (Fig. 2b) and phosphate (Fig. 2c) at P5 indicated a possible release of the anions and leachable phosphate from compartment C and D within the column.

The same trend was observed in the third run except that while the nitrate was completely depleted, the nitrite began to build up to the fifth sampling point (Fig. 2c). This development indicated the possibility of oxygen infusion, nitrate reduction to ammonia^30^ and/or incomplete denitrification due to shorter retention times. This informed our decision to lower the flow rate from 3.15 mL min^−1^ to 2.5 mL min^−1^ to increase the residence time.

With the adjustment of the flow rate, however, a further shift of the nitrite peak was noticed to P_4_ instead of P_3_ (Fig. 2d). This indicates an improvement in remediation because from a maximum concentration of 182 ppm (32 mg L^−1^ which is greater than three times the maximum permissible limit of 50 mg L^−1^), the nitrate and nitrite were both successfully remediated to 0.0 and 0.6 mg L^−1^ respectively. In concordance with previous research, the analysis using distilled water revealed the influence of the flow rate,^15^ nature of the carbon source and timing of the process on the attenuation pattern.^9,31^

#### 3.1.2 Phase II – well water

**Fig. 3:**
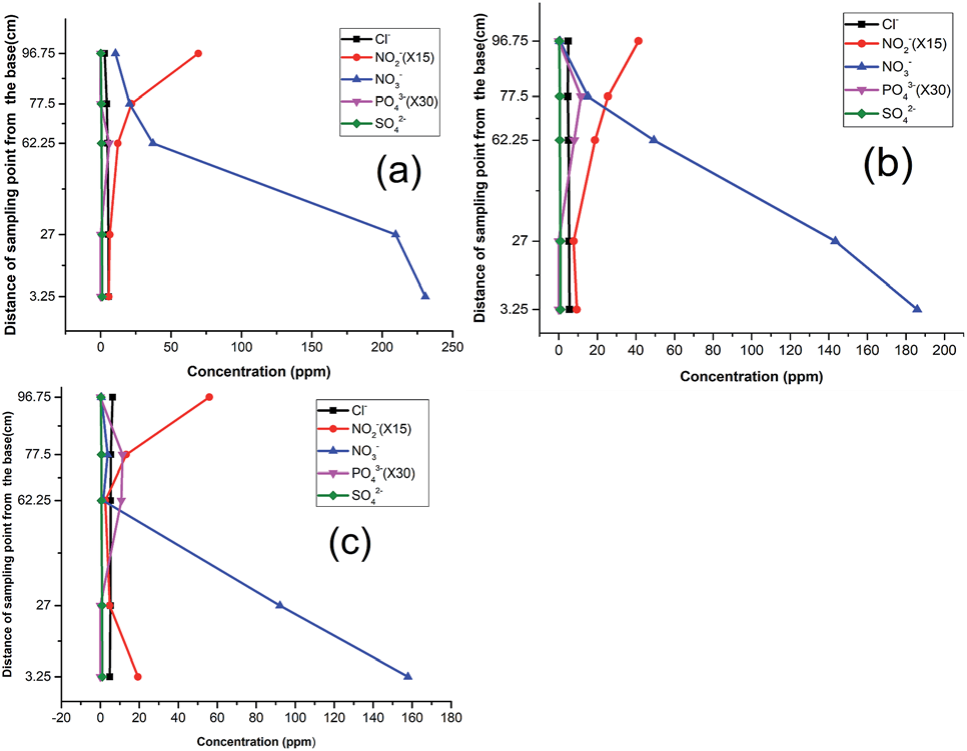
Response of NO_3_^−^ spiked well water: (a) 1^st^ run, (b) 2^nd^ run, (c) 3^rd^ run.

The attenuation pattern of the nitrate-spiked well water (230 ppm) was similar to that of the distilled water, wherein the fastest decline of nitrate concentration was observed between P_2_ and P_3_ (sawdust chamber). However, nitrite was observed to build up from P_3_ to P_5_ (Fig. 3a). Unlike in the preceding phase, the inverse relation between nitrate and nitrite here was not pronounced. This is attributed to the matrix effect of the natural water as it is reported to support more redox processes in the presence of natural concentrations of species such as O_2_, H_2_S, Fe^2+^, CH_4_, SO_4_^2−^*etc.*^32^ Thus, the microbial community is bound to find the groundwater more suitable for metabolic activities than distilled water. The observed changes in the third compartment could be attributed to the microbial response to the different environment (pH) and hence the nitrite production.^33,34^ A similar phenomenon has been found to occur in culture under stressed conditions.^35^

As one of the reasons for the above response, the high concentration of nitrate in the influent (230 ppm) was reduced to 185 ppm (Fig. 3b). However, no significant improvement was recorded because the nitrite still rose at P_5_. A curious inverse relation between chloride and nitrite suggested that these were favorable conditions for eluting adsorbed chloride from the activated carbon. This was corroborated by the observed decrease in pH around the media, and hence indicated a slowed denitrification process.^36^ A further reduction of the influent nitrate concentration to 157 ppm in the third run did not yield improved results either. As in the previous run, phosphate emerged at P_3_ and P_4_, affirming the release of dissolved organic phosphate from the wood media (Fig. 3c).

**Fig. 4:**
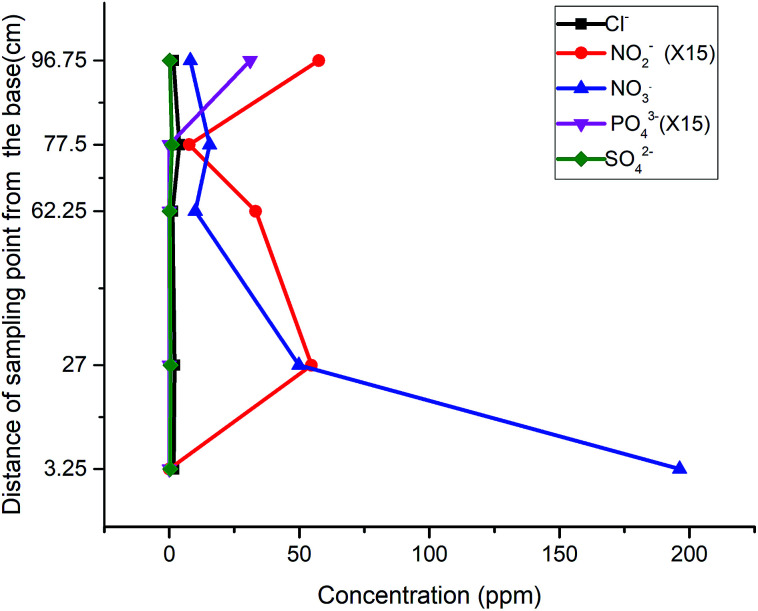
First run of nitrite-spiked well water in column containing lower amounts of carbon-based constituents.

Considering the surge of leachable products in the column, adjustment of column material composition was deemed necessary and hence made to lower the amounts of carbon-based constituents across the compartments with 15%. The concentration of the nitrate was then raised to 196 ppm to observe the trends at that ratio. The result indicated a sharp decline of nitrate concentration with an accompanying surge of nitrite production (Fig. 4). Similar to the previous runs, the nitrite values indicated a build up at P_5_, signaling the need for further modification.

#### 3.1.3 Phase III – modified column

Although the concentrations of nitrate and nitrite fell within the maximum permissible limits, the elevated concentrations of nitrite near the threshold limit value of 3 mg L^−1^ were uncomfortable with respect to achieving the remediation goal. Two factors were therefore considered, and adjustment was made to the column composition. In order to regulate the pH and buffer for alkalinity,^37^ and to provide dissolved inorganic carbon in the form of CO_2_ for sulfur related autotrophic or hydrogenotrophic denitrification,^5,38^ varying proportions of lime (CaCO_3_) were added to the different compartments as described below. The buffering action was expected to address the effect of pH fluctuation from one medium to another, which might upset the stability of the microbial community and affect its performance. This led to phase III of the experiment with the creation of a fresh lime-embedded composition. The first compartment (A) of the column consisted of soil, date seed, *Moringa oleifera* seed and limestone in the ratio of 115.4 : 50.5 : 1 : 24.3 while the second compartment (B) consisted of soil, wood chips, sawdust and limestone in the ratio of 32 : 3 : 1 : 5.2 (w/w). The third compartment (C) represented the final stage providing a carbon source with the constituents of soil, date seed and limestone in the ratio 8.3 : 1.7 : 1, while the fourth compartment (D) had soil and activated carbon in the ratio of 1 : 1.6 (w/w).

**Fig. 5:**
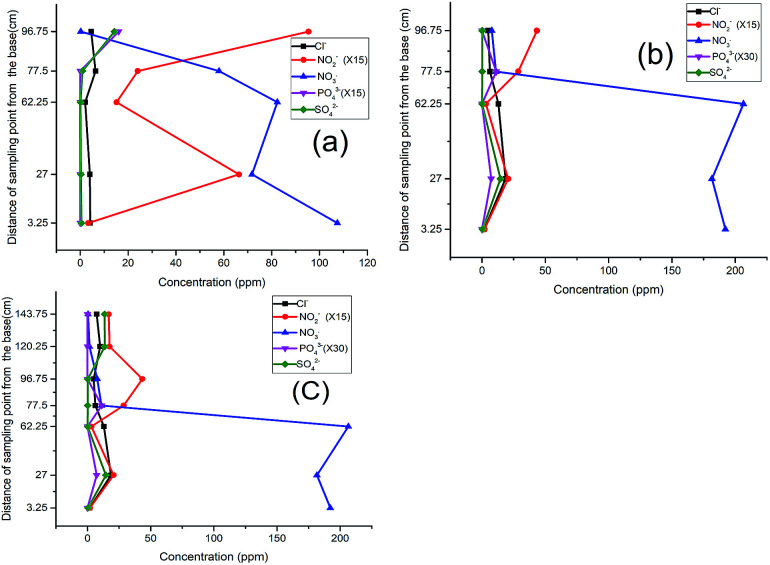
Nitrite-spiked well water in the modified column: (a) 2^nd^ run, (b) 3^rd^ run, (c) 4^th^ run.

The first run of the modified column revealed a skewed pattern for both nitrate and nitrite, with nitrate gaining fresh momentum in the sawdust chamber and nitrite rising much higher in the last compartment (Fig. 5a). The former could be attributed to leachable nitrate in the second compartment while the latter was attributed to possible incomplete denitrification or elution of adsorbed nitrite from the activated carbon in the previous run.

The influent concentration was raised to 196 ppm for the second run, and a continuous flow of the influent through the column revealed a much lower concentration of SO_4_^2−^ at P_4_ and P_5_ in the third run, justifying the assumption of its elution from the activated carbon (Fig. 5b). The recurrence of an elevated concentration of nitrate at P_3_ and its subsequent drop at P_4_ confirmed the leaching property of the sawdust chamber. Nitrate attenuation was inversely matched to that of nitrite but as the time passed by, the amount of nitrite generated at the exit reduced to less than 50% of that generated in the previous test, such that the concentration dropped from 6.36 to 2.89 ppm.

Further tests to determine the impact of the packing structure on the attenuation pattern of the parameters down the gradient attested to the efficiency of the reactive media over a short distance: when the effluent of the above assembly was used as the influent of a whole soil column, the concentrations of nitrite and nitrate were reduced to 98% and 60% respectively (Fig. 5c). This excellent attenuation capacity of the soil was however higher than that observed when a nitrate spiked sample was passed directly through a soil column, suggesting that the attenuation capacity of soils is improved when the process of remediation has begun for the influent.

**Fig. 6:**
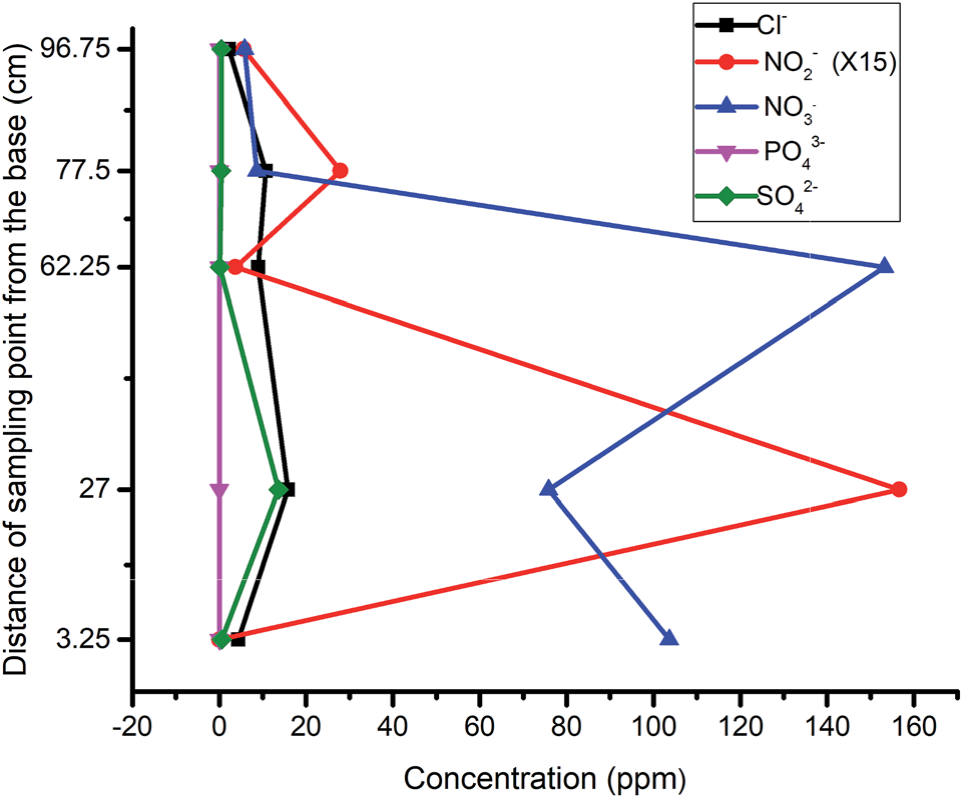
Third run of nitrite-spiked well water in the modified column.

Having observed the trend of descending nitrite concentration within the gate and down the gradient, another run of influent with a lower nitrate concentration of 103 ppm was tested to determine whether the assembly could be applied without further attenuation down the gradient. The result revealed a reduced concentration of nitrate to 5.81 ppm and nitrite to 0.37 ppm (Fig. 6). This final test depicted the threshold limit concentrations of the reactive media, when natural attenuation capacity was ruled out. Also, the results obtained throughout the test indicated that the release or leaching of organic by-products and other inorganic radicals is dependent upon the degradation process taking place, which progresses over time.

The general pattern of pH attenuation indicated that the pH response in distilled water was higher and the separation was better than those in groundwater. This may be due to the complex chemical matrix in groundwater, which is not present in distilled water. It was also observed that in both groundwater and distilled water, an elongation of the experimental period led to an improvement, shown by the rise in pH which reveals the significance of a microbial incubation and adaptation period.

**Fig. 7:**
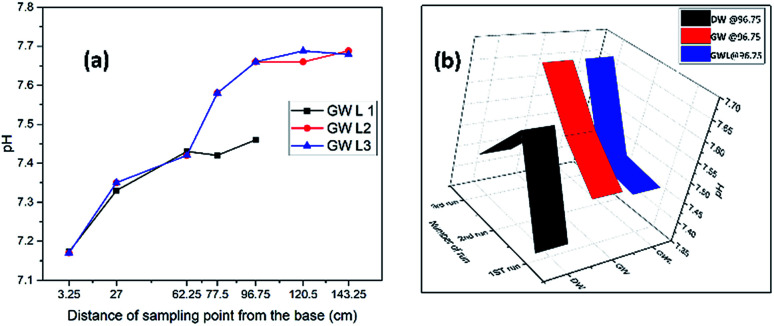
pH attenuation pattern for (a) the 1^st^, 2^nd^ and 3^rd^ runs of groundwater with lime (GWL), (b) the last sampling points (96.75 cm) for the 1^st^, 2^nd^ and 3^rd^ runs of distilled water (DW), groundwater (GW) and groundwater with lime (GWL).

A plot of pH attenuation at the exit sampling points (97.65 cm and 143.25 cm) (Fig. 7b) for the first, second and third runs using distilled water, groundwater and groundwater with lime indicated a progressive increase in pH and a stabilized pattern for the groundwater with lime. The pattern of increasing pH was observed in the distilled water sample at the latter stage. The groundwater without lime however showed a pattern of descending pH with irregular distribution. This shows the significance of lime in improving and stabilizing the pH, as can be seen in Fig. 7a, where successive runs of the experiment in groundwater with lime gave a rise in pH.

### 3.2 Effect of carbon source composition

It was observed that the reduction of total organic by-products by the system was best achieved through down gradient attenuation. Starting with the initial TOC value of 3.55 ppm in the influent (at P_1_) and a maximum TOC concentration of 40.35 ppm at P_3_, a carbon buildup of 18.77 ppm was recorded at the exit of the soil column. This represents an 11-fold increase in carbon input by the materials and a 53% reduction of the carbon by the system. Because of this, and the fact that the NO_3_^−^ concentration dropped from 192.14 ppm to 11.09 ppm at the exit of the first column and finally to 1.56 ppm at the exit of the second (soil) column, it is safe to assert that unlike similar remediation alternatives, our laboratory scale sequence multi-barrier reactive media has the advantage of remediating 94.2% and 99.2% of the target pollutant (NO_3_^−^) within and outside the system (down the gradient) respectively. Also, the system generated NO_2_^−^ (within the denitrification process) whose concentration was three times the threshold limit value of 3 ppm (10.4 ppm) (Fig. 6). This concentration was scaled up to 156 ppm (10.4 ppm × 15), but the final NO_2_^−^ concentration dropped to 5.07 ppm, and so the system is deduced to offer a 96.4% reduction of nitrite.

**Fig. 8:**
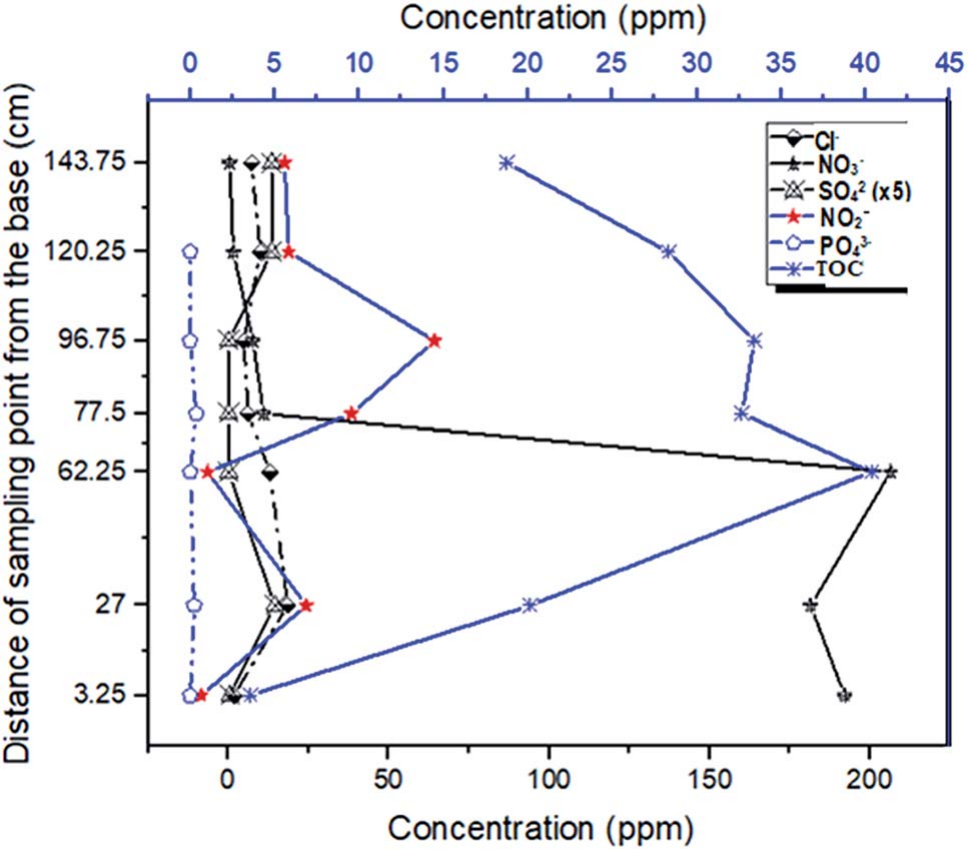
Pattern of Total Organic Carbon (TOC) distribution across the sampling points.

The relatively low attenuation capacity for organic substituents within the set-up was attributed to the low flow rate, various carbonaceous matrixes (labile and recalcitrant) and the short distance in the system (as compared to that of an aquifer). The low flow rate leads to the dissolution of more organic substances out of which the labile fraction is mostly utilized by the bacteria, generating incomplete denitrification with concomitant liberation of nitrite. As the nitrate is exhausted, the rate of degradation slows down, and since denitrification is a major source of organic matter removal,^39^ this means that the dissolved complex organic components take longer to be completely degraded.^9^ A further observation of the effluent behavior in the simulated pristine aquifer condition (in a soil column) revealed a gradual, but effective downward trend in attenuation of the organic constituents generated by the media at the gate (Fig. 8).

### 3.3 Surface characterization techniques

#### 3.3.1 FESEM-EDX

**Fig. 9:**
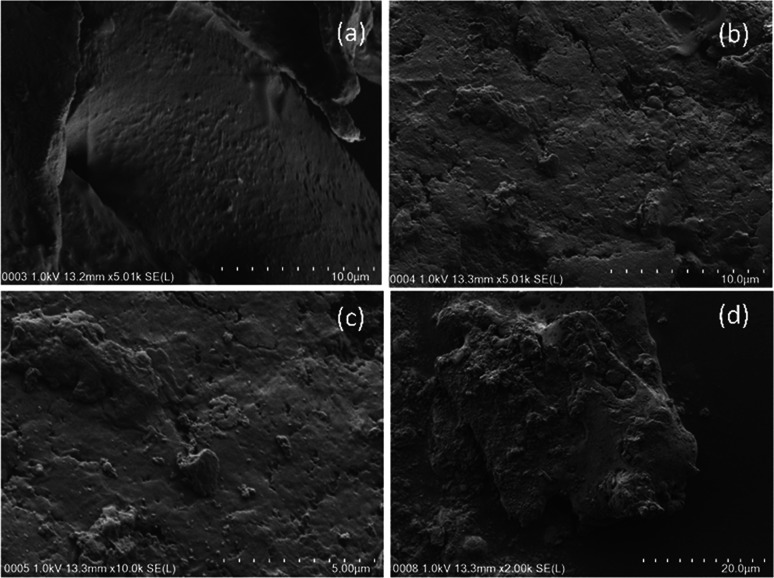
FESEM images of (a) fresh date seed (10 μm), (b) used date seed (10 μm), (c) used date seed (5 μm), (d) used date seed (20 μm).

**Fig. 10:**
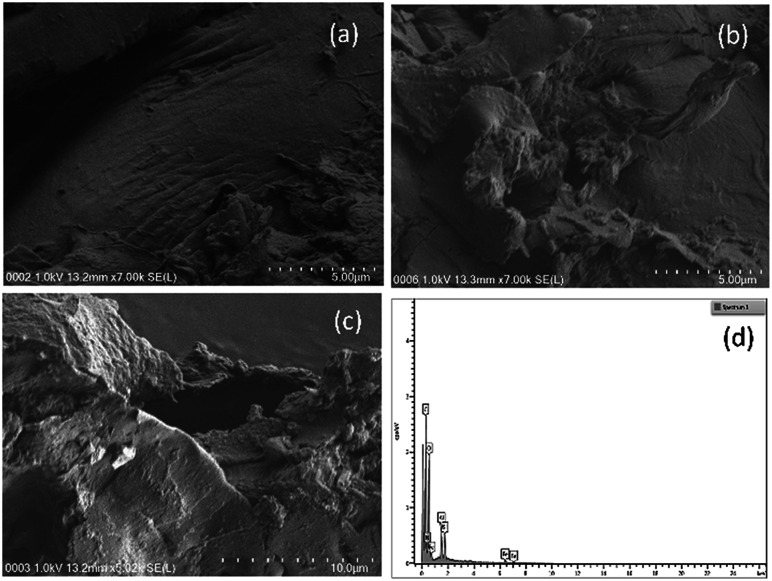
FESEM images of (a) fresh wood chips (5 μm), (b) used wood chips (5 μm), and (c) used wood chips (10 μm). (d) Elemental mapping from EDX of used date seed (25 μm).

FESEM-EDX analysis indicated that there was preferential decomposition among the materials. The date seed maintained its surface texture relatively well after the column analysis and only exhibited a few cracks and tiny pores (Fig. 9b and c), while the wood chips were deformed with wider openings across their surfaces (Fig. 10b and c). The distortion was conspicuous in both the 10 μm and 5 μm images. Fragments of the weakened surface were also evident in the images of wood chips after the column experiment (decomposition).

The image of the date seed taken at 2000× magnification (20 μm) revealed fragmental deformation of both the size and the surface at a lower scale, depicting an image of cracks associated with the crushing activity rather than the decomposition (Fig. 9d). This may be associated with the composition of date seed, which has been reported to consist of hemicelluloses (23%), lignin (15%) and cellulose (57%).^40^ It was also observed that the medium magnification image exposed more of the elements for EDX mapping. This was evident from the EDX analysis of the 5000× magnification image of the wood chips (result not shown).

The elemental mapping of the fresh wood chips indicated that carbon was present in the highest % by weight (61.12%) followed by oxygen (31.79%) and then nitrogen (7.09%) (Table 1). After subjecting the wood chips to the column test, however, new elements emerged with a concomitant decrease in the % weight of carbon to 42.96% and increases for nitrogen and oxygen to 7.58% and 33.36%, respectively (Table 1).

**Table 1:** EDX elemental mapping for fresh and used wood chips (10 μm)

S/N	Element	Wood chips before (10 μm), weight%	Wood chips after (10 μm), weight%
1	C	61.12	42.96
2	N	7.09	7.58
3	O	31.79	33.36
4	Al	—	3.85
5	Si	—	4.13
6	Fe	—	8.12
	Total	100.00	100.00

While the decline in mass of the carbon (30% depletion) was understandable on the basis of dissolution and decomposition, the rise of nitrogen might indicate possible leaching or some adsorbed portion of the nitrate spiked influent, or both. The elements of Al, Si and Fe emerging post-column accounted for 3.85, 4.13 and 8.12%, respectively. Here, while a possible leaching of aluminum was envisaged, much of the Fe and all of the Si would have been derived from the soil embedded in the column.

In contrast to the wood chips, the elemental mapping of fresh date seed by EDX revealed four elements: carbon, oxygen, potassium and nitrogen, with % weight distributions of 74.36%, 20%, 5.37% and 0.27% respectively (Table 2). After the column test, the weight% values changed to 67.85%, 22.36% and 9.79% for carbon, oxygen and nitrogen respectively (Table 2). This reaffirmed the trend of carbon depletion (with a 6.51% reduction over the given period) and the appreciation of oxygen and nitrogen, as experienced in the wood chips.

**Table 2:** EDX elemental mapping for fresh & used date seed (10 μm)

S/N	Element	Fresh date seed (10 μm), weight%	Used date seed (10 μm), weight%
1	C	74.36	67.85
2	N	0.27	9.79
3	O	20.0	22.36
4	K	5.37	
	Total	100.00	100.00

Upon stretching the area of magnification of the used wood chips to 25 μm scale (Fig. 10d), additional elements were detected. Among them were Ca (at low percentage), Na and Fe. While Ca has been reported in date seed,^41^ Fe was reported to have been isolated from an extracellular substance with a strong adsorption for iron in a decaying wood.^42^ Besbes, *et al.*^43^ reported only 0.0023% Fe in date seed. The emergence of these elements after the column test implied that some of them were leached or sequestrated as the decomposition progressed.^41^

Finally, it can be deduced from the FESEM images of the surfaces of the wood chips and date seed that the wood chips were more susceptible to biological decomposition than the date seed. It is also evident from the EDX results that the 30% reduction in carbon in the wood chips was by far more than the 6.51% reduction of carbon in the date seed over the same period. In both cases, nitrogen and oxygen appreciated significantly, indicating the effect of the nitrate-spiked influent water and the redox environment on the degradation processes.

#### 3.3.2 Fourier-transform infrared spectroscopy (FTIR)

**Fig. 11:**
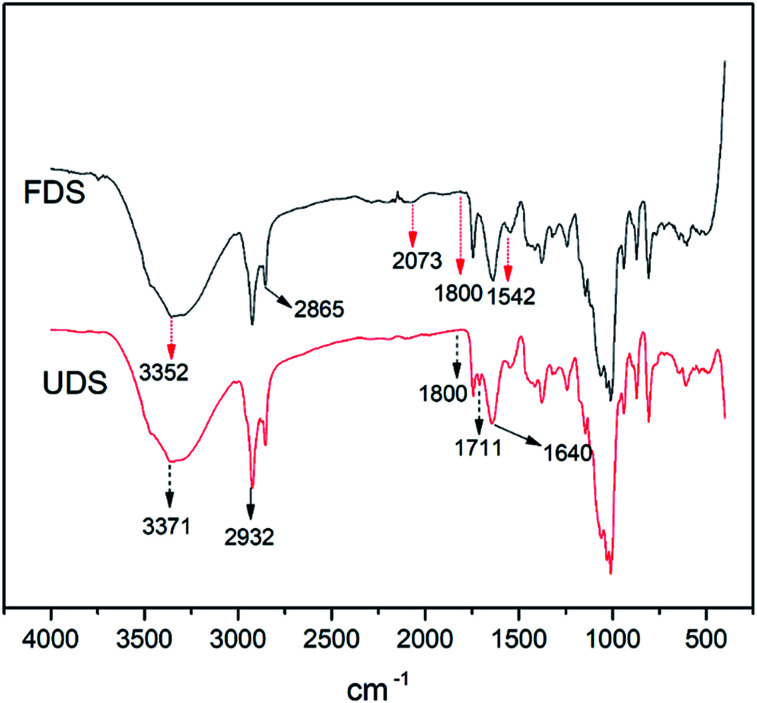
FTIR spectra of fresh date seed (FDS) and used date seed (UDS).

The same samples of date seed and wood chips obtained before and after the column test were subjected to FTIR analysis to observe changes in chemical functional group formation and removal. Analysis for the date seed shows that distortion in the absorption intensities occurred in the region 2250–750 cm^−1^. In line with a previous research finding, there was no change in the bands observed at 2932 cm^−1^ and 2865 cm^−1^ assigned to the asymmetric and symmetric vibration of CH_2_ and CH_3_, or the band at 1640 cm^−1^, which fell at the borderline of 1650 cm^−1^ assigned to the carbonyl stretching vibration of amide due to the double bond.^44^ It was hereby inferred that this stability denoted resistance to decomposition, unlike for the wood chips. However, in the fresh date sample, there was a notable peak at 2073 cm^−1^ indicating (N=C=S) absorption due to isothiocyanate stretching which was missing in the decomposed date seed. Conversely, the peak at 1711 cm^−1^ in decomposed date seed, assigned to carbonyl C=O absorption due to carboxylic acid stretching, was missing in the fresh seed (Fig. 11). This indicated a redox situation consisting of bond formation and breakage in the process of decomposition.

In line with a previous research finding on date seed, all the samples manifested weak bands at 1800 cm^−1^, indicating the presence of aromatic compounds.^45^ In addition, the strong and broad band for –OH absorption of alcohol stretching (3550–3200 cm^−1^) was present in both fresh and decomposed seed. In addition to the carboxylic acid group identified in the decomposed date seed, this could explain why decomposed products always recorded a higher oxygen content in the EDX results than the fresh products.

**Fig. 12:**
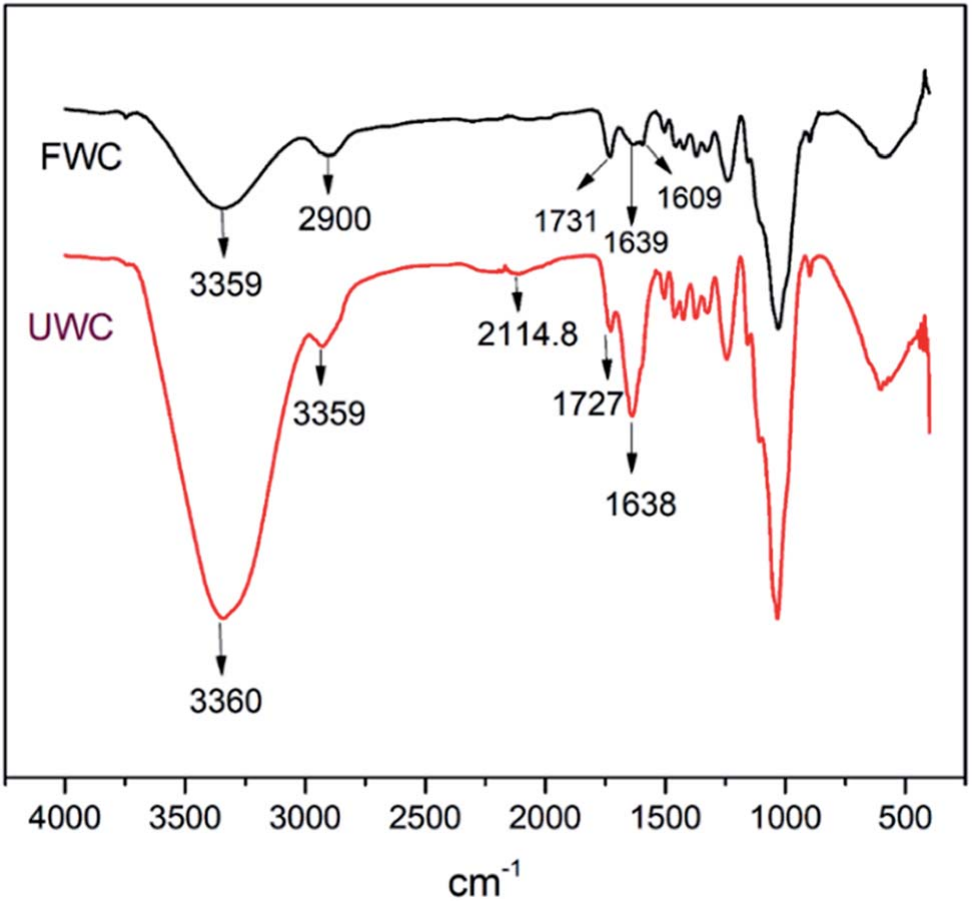
FTIR spectra of fresh wood (FWC) and used wood (UWC).

The wood chips spectra manifested distortions in intensities that occurred in the region 2115–1250 cm^−1^ (Fig. 12) which is a wider region wider than that obtained by Pandey^46^ using transmission and drift methods of FTIR. However, the manifestation of a C–H stretching absorption around 2900 cm^−1^ obtained in the research by Pandey coincides better with the results for the fresh wood than for the used wood in this study.

The other signals observed were of the same skeleton, but from different functional groups. Here, the signal at 1727.86 cm^−1^ denoting the carbonyl C=O absorption of aliphatic ketone stretching was observed in decomposed wood chips while signal at 1731.64 cm^−1^ for C=O absorption of β-unsaturated ester stretching was observed in fresh wood chips.

The spectra displayed significant differences in absorption bands between the fresh wood and decomposed wood for alkyne stretching (C≡C absorption, falling within the range of 2140–2100 cm^−1^). The protruding weak signals within the vicinity of the stretching range of monosubstituted alkynes at 2114.89 cm^−1^ in the decomposed wood attested to that.

In the same vein, the aromatic skeletal vibration band at 1609 cm^−1^ for the fresh wood chips diminished for the used wood chips, indicating a possible ring opening reaction during the decomposition. However, the intensity of the band at 1638 cm^−1^ (C=C absorption due to alkene stretching) in used wood chips was higher than the corresponding signal for fresh wood chips, supporting the argument that bond breakage was promoted in the decomposition process. These changes indicated the occurrence of reduction at that particular site. Finally, the strong and broad band for –OH absorption of alcohol stretching (3550–3200 cm^−1^) was present in both samples but it was more prominent in the decomposed wood chips than in the fresh woodchips. In summary, the process of decomposition entailed multiple redox reactions at different sites and, depending on which bond or functional group was affected in the process, different transformations were evident in this study.

### 3.4 Rates of decomposition

The commonly used single-exponential model (eqn (2)) was considered first in this study and the relative decomposition rates, are expressed as percentages. In line with previous findings,^47^ readings obtained from the observation of the decomposition process revealed that the initial rate and the late stage decomposition for both date seed and wood chips were very slow, indicating a preliminary acclimatization period. Also, an inverse relation emerged between the date seed and wood chip on the seventh month of the study but the rate of degradation was faster in wood chips than in date seed.

2 *X* = *X*_o_e^−*kt*^

**Fig. 13:**
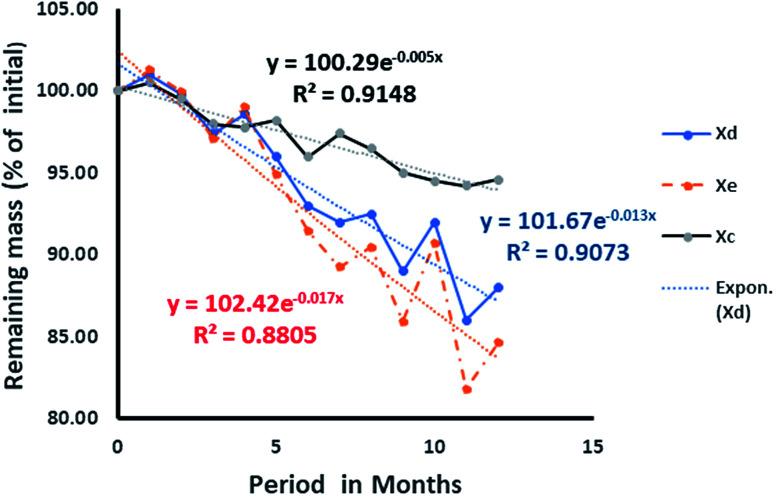
Rate of degradation of date seed: Xd is the entire date, Xc is the cover wall of the seed, and Xe is the deduced degradation pattern of the endosperm.

**Fig. 14:**
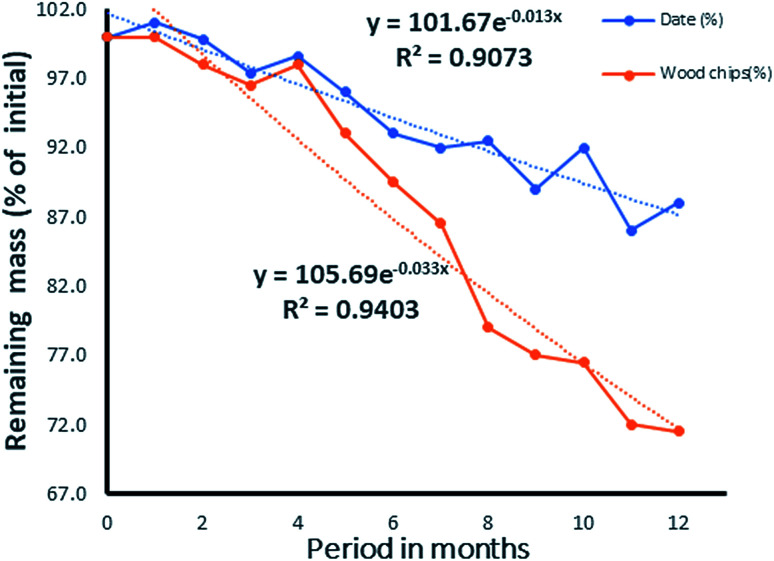
Comparative rates of degradation of date seed and wood chips in the same column.

While the decomposition of wood chips (Fig. 14) indicated a possible steady rate at the eleventh month, that of the date seed appeared slower and inconsistent. In the latter, the observation was attributed to the varying decomposition rates of the endosperm and the hard wall cover of the date seed, as shown in Fig. 13. From the values of *R*^2^ for both the date seed and the wood chips, it could be deduced that the exponential rate model fitted the decay pattern of the materials. The interpolated degradation pattern of the date endosperm (Xe) had an *R*^2^ value of 0.8805, but the date seed, date hard cover and wood chips had good *R*^2^ values of 0.9073, and 0.9148 and 0.9403, respectively. While the *k* values depicted the degree of the rate of degradation, the distribution patterns showed the differences in degradation potential. The degradation pattern of the endosperm (interpolated values) dictated the degradation pattern of the entire seed and hence, a similar distribution was observed for the date seen (Xd) and endosperm (Xe) (Fig. 13).

The initial surge in mass observed in the first month for the date seed could be attributed to the binding capacity of the soft endosperm surface to dissolved organic debris from sawdust within the column. It is pertinent to observe that the sharp decline in the date seed plot began at the fourth month of the column operation and at that moment, the degradation rate of the date cover began to decline.

The same observation could be made from the sixth month through to the eighth month, where every slight loss of mass of the date seed was accompanied by slower degradation of the date cover and *vice versa*. In the same vein, from the seventh month onwards, whenever the date seed cover mass declined, the percentage mass loss of the interpolated endosperm matched more closely with that of the entire date seed and *vice versa*. We might infer that this is the period in which the date cover started to affect the degradation rate of the entire seed and hence becomes the rate determining substrate.

This differential influence of the date cover on the degradation potential might explain why the date cover tended to indicate a stabilising pattern from the ninth month onward while the date seed was yet to show any signs of stability one year after the beginning of the experiment. This also clearly indicated the presence of a recalcitrant component within the seed. Hence, we considered the double exponential model (eqn (3)):

3 *X* = *A*e^−*k*_1_*t*^ + (1 − *A*)e^−*k*_2_*t*^

where *X* stands for the total mass remaining after time *t*, *A* is the labile portion of the material under review and (1 − *A*) is the recalcitrant portion. To determine *X* through the above exponential models, the values of the results obtained at the seventh month were substituted into both eqn (2) and (3). With values of *X*_o_ = 1.11 g, *k*_1_ = 0.01, *k*_2_ = 0.005, *t* = 0.5833 (7/12) and *A* = 0.66 g, substitution of the values into the equations gave the following results:

Single exponential*X* = *X*_o_e^−*kt*^, *X* = 1.1035

Double exponential function*X* = *A*e^−*k*_1_*t*^ + (1 − *A*)e^−*k*_2_*t*^, *X* = 1.099

With a differential margin of 8.05% and a 7.64% deviation from the experimental value of 1.02 for the single exponential and double exponential results respectively, it could be deduced that the double exponential model gave a better fit than the single exponential model as it was closer to the experimental value.

Moreover, judging from the value of the rate constants alone, a comparative analogy of differential life spans could be drawn for the wood chips and date seed. Using the rate constant to determine the half-life *t*_(0.5)_, it follows that;*t*_(0.5)_ = −ln(0.5)/*k* = (0.693)/*k*

Given the respective values of *k* for the date seed and woodchips, the half-lives would be
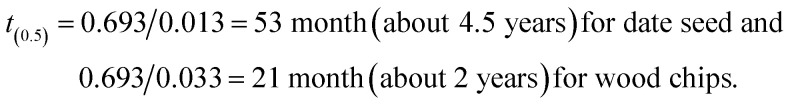


These suggest a life time stretching to 9 years for 1 g of date seed and a life time of 4 years for 1 g of wood. The life time obtained here is similar to that for the low density woods reported by Mackensen *et al.*^26^ Depending on the quantity and quality of the carbonaceous material used, the lifetime could be several times higher than that obtained here. The advantage of the date seed is due to the slow degradation rate of the cover wall, which contains mannan. Since the above half-life was calculated based on exhausting 95% of the material, the 5% that would remain after 9 years would be the cover wall. Going by its “*k*” value, the half-life of the cover would be 138.6 months, which is equivalent to 12 years. The lifetime of the date seed could then be deduced to be 24 years under such weathering conditions. With this, it could be deduced that the date seed would last as much as six times longer than a wood chip before complete degradation. Though the process could be slow in the date seed, it would assure a constant supply of carbon for a longer period, serving the most pressing need of current *in situ* remediation technology.

## 4 Conclusions

The laboratory column analysis designed and arranged in line with the hypothetical packing structure of gate materials indicated a clear variation of response to denitrification due to a matrix effect: groundwater proved more complex to deal with than distilled water. The efficiency of the column set-up was found to be dependent upon the ratio, composition, flow rate and initial concentration of NO_3_^−^ in the influent. The introduction of limestone into the first, second and third compartments in the column was found to be useful in stabilizing the pH and lowering the NO_2_^−^ build-up in the system.

The two surface characterization techniques employed for the carbonaceous substances (FESEM-EDX and FTIR) indicated a variation in morphological responses between the materials (date seed and wood chips) and within each material before and after the decomposition process. By considering the differences between the holes and cracks on the surface of the materials before and after decomposition, it was deduced that wood chips were more susceptible to decomposition than date seeds. The EDX results and FESEM analysis suggested that the percentage of carbon loss in wood chips after decomposition was three times higher than that lost in date seed decomposition. The FTIR analysis of the used wood chips indicated more reduction changes, affirming the susceptibility of the wood to consumption by microbes, and hence, it was more prone to decomposition than date seed.

The above observations tallied with the results of the study on the rate of decomposition, as the respective half-lives of 2, 4.5 and 12 years were deduced for 1 g of wood chips, 1 g of date seed and 0.37 g of date cover, respectively. The lifetime of the date seed could then be deduced to be 24 years. Under weathering conditions, date seeds assure a constant supply of carbon for a longer period, serving the most pressing need of current *in situ* remediation technology. It could be further deduced that the date seeds would last as much as six times as long as wood chips before complete degradation. Finally, the double exponential model was found to fit the degradation of date seeds better than the single exponential model in expressing the decay rate.

It could therefore be concluded that in addition to the successful remediation capacity of the multi-layered sequential treatment barrier, the physical and morphological analysis obtained from the surface characterization techniques revealed a preferential consumption of one carbon source over another. This preferential consumption by the degrading microorganisms would surely cause delay in the degradation process and hence increase the lifespan of the gate materials. With a lifespan that is as much as six times longer than that of the wood chips, date seed has the potential to replace the preferred wood chip in the remediation industry.

It is recommended that more effort be put into the utilization of biodegradable seeds with higher carbon content in PRB gate materials. This is because the seeds with high lignin content are an environmental liability, consuming energy and resources for their disposal. It is also recommended that the described composition and order of placement be utilized in the field for the long-term treatment of pollutants.

## Conflicts of interest

There are no conflicts to declare.

## Supplementary Material
